# Fatal Delayed Cardiac Tamponade Following Atrial Fibrillation Ablation: A Case Report

**DOI:** 10.7759/cureus.75698

**Published:** 2024-12-14

**Authors:** Sylusha Gadipudi, Yashitha Chirumamilla, Philip J McDonald

**Affiliations:** 1 Internal Medicine, Hurley Medical Center, Flint, USA

**Keywords:** acute pericardial effusion, atrial fibrillation ablation, cardiac catheter ablation, cardiac tamponade, delayed postoperative cardiac tamponade, pericardial tamponade, pericardial window

## Abstract

Catheter ablation procedure for symptomatic atrial fibrillation is an established treatment. Cardiac tamponade is one of the several complications associated with atrial fibrillation ablation. We present the case of a 60-year-old male with a past medical history of end-stage renal disease (ESRD) on hemodialysis, hypotension on midodrine, atrial fibrillation status post-ablation a week prior, and a cerebrovascular accident who presented to the emergency department with complaints of weakness, nausea, vomiting, confusion and some syncopal episodes for the past few days. He denied chest pain, dyspnea, and a history of alcohol use. On arrival at the emergency department, the patient was hypotensive and tachycardic. Laboratory evaluation revealed elevated liver enzymes and troponins. The electrocardiogram revealed sinus tachycardia. Chest X-ray showed low lung volumes with a left retrocardiac opacity and cardiomegaly. Given the patient's hypotension requiring vasopressor support, elevated troponins, and a recent cardiac procedure, an echocardiogram was done, and it revealed a large posterior pericardial effusion adjacent to the left ventricle. Tamponade was suspected and the patient was taken for an emergent pericardial window.

Intraoperatively, a perforation of the right ventricle was found with a large area of surrounding necrotic and ischemic tissue. There was a significant hemorrhage with the opening of the pericardium, which could not be controlled due to the large area of non-viable cardiac tissue. The patient went into cardiac arrest with pulseless electrical activity. Open cardiac massage was performed but the return of spontaneous circulation could not be achieved and the patient was declared deceased. Pericardial effusion with tamponade is the complication of cardiac ablation associated with the highest mortality. An echocardiogram is a fast and reliable way to confirm the diagnosis of a tamponade. Delayed cardiac tamponade should always remain a differential diagnosis when a patient has recently undergone an ablation procedure given the potential fatality.

## Introduction

Catheter ablation procedure for symptomatic atrial fibrillation is an established treatment, which has become increasingly popular in recent years. There are several complications associated with atrial fibrillation ablation; cardiac tamponade is a serious one among them. It is one of the major causes of mortality associated with surgical procedures. Cardiac tamponade can be divided into two types, depending on when it occurs: early or acute, occurring within 48-72 hours after cardiac surgery, and subacute or delayed, occurring two to three days later without obvious clinical signs. A case-control study was conducted to identify factors associated with delayed cardiac tamponade (DCT) after cardiac surgery. It concluded that factors such as full pre- or post-operative anticoagulation, surgery other than coronary artery bypass graft, and need for red blood cell transfusion. Surgical reintervention in the first 48 hours following surgery is a predisposing condition for the appearance of subacute or delayed tamponade [1]. Of the previously mentioned, our patient possessed only one risk factor, preoperative anticoagulation.

## Case presentation

We present the case of a 60-year-old male with a past medical history of end-stage renal disease on hemodialysis, hypotension on midodrine, atrial fibrillation status post-ablation a week prior, cerebrovascular accident presented to the emergency department with a chief complaint of weakness and vomiting for the past few days, following the procedure. He reported intermittent confusion, dizziness, and a few syncopal episodes. He denied chest pain, dyspnea, and a history of alcohol use.

On arrival at the emergency department, the patient's blood pressure was 97/65 mm Hg. He was tachycardic with a heart rate of 106 beats per minute. Physical examination revealed equal breath sounds bilaterally, a normal S1 and S2 with no murmurs, and no other positive findings. Remarkable laboratory findings are mentioned in Table 1.

**Table 1:** Initial laboratory evaluation obtained upon arrival to the emergency department.

Laboratory test	Value	Reference range
Hemoglobin	12.7 g/dL	13.5-17.5 g/dL
White blood cell count	8.8 K/μL	4-10.8 K/μL
Platelet count	147 K/μL	130-430 K/μL
Aspartate transaminase	1370 U/L	0-40 U/L
Alanine transaminase	705 U/L	7-40 U/L
Gamma-glutamyl transferase	60 U/L	1-30 U/L
Peak troponin	0.0360 ng/mL	<= 0.040 ng/mL

**Figure 1:**
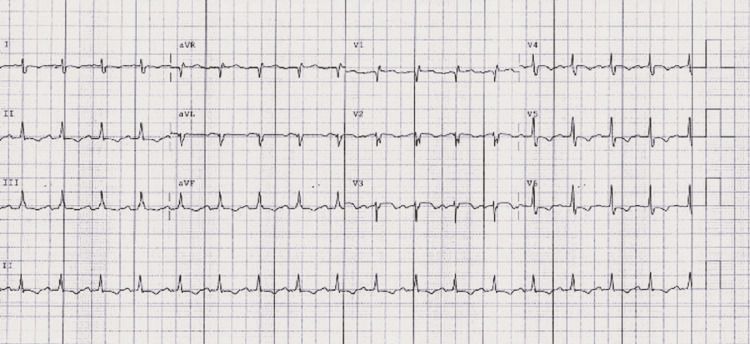
Initial electrocardiogram obtained upon patient's arrival depicting sinus tachycardia with no acute ischemic changes.

An electrocardiogram (ECG) revealed sinus tachycardia without any acute ischemic changes (Figure 1).

**Figure 2:**
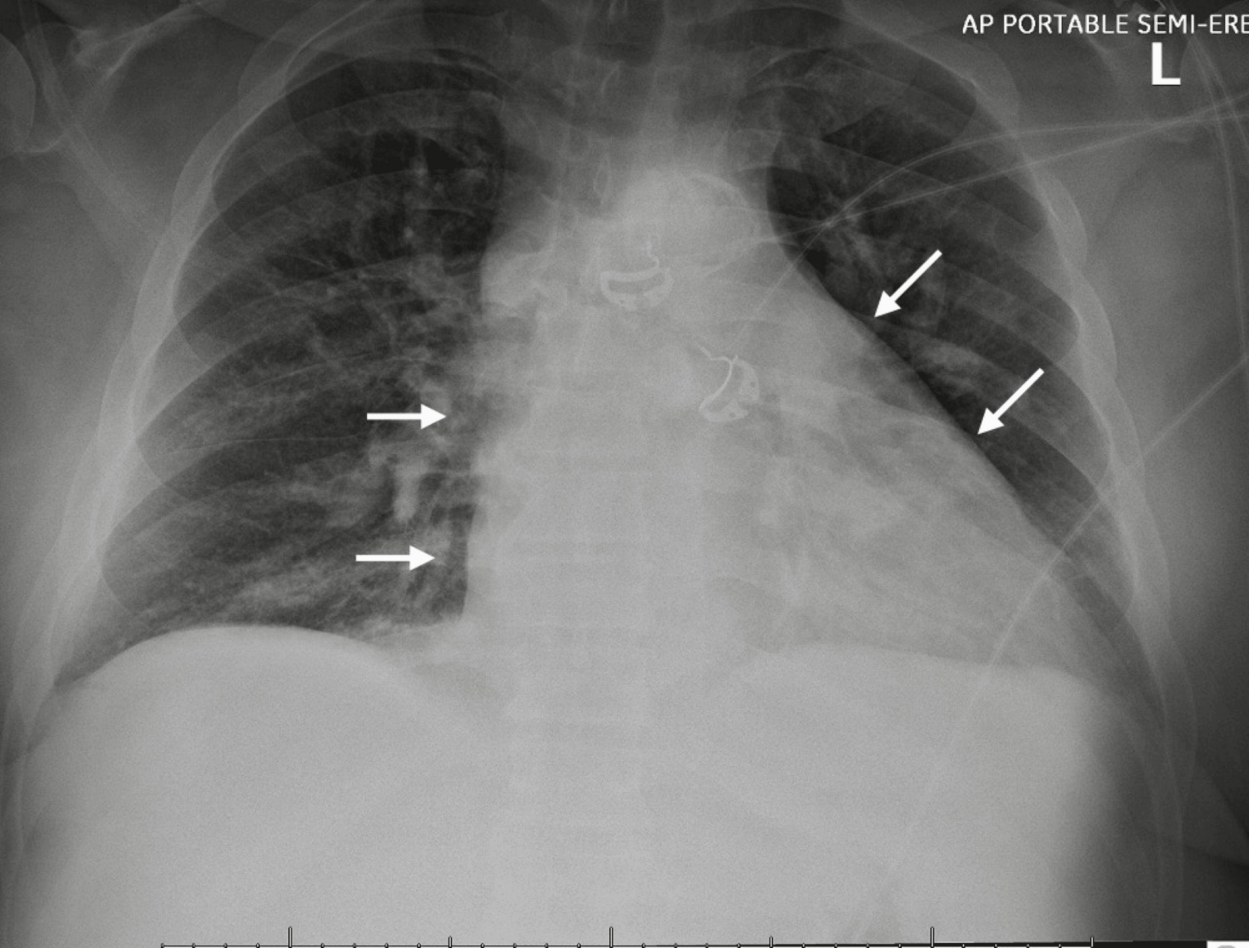
Chest radiography depicting cardiomegaly with an enlarged cardiac silhouette as shown by the white arrows.

Chest X-ray showed low lung volumes with a left retrocardiac opacity and cardiomegaly (Figure 2).

**Figure 3:**
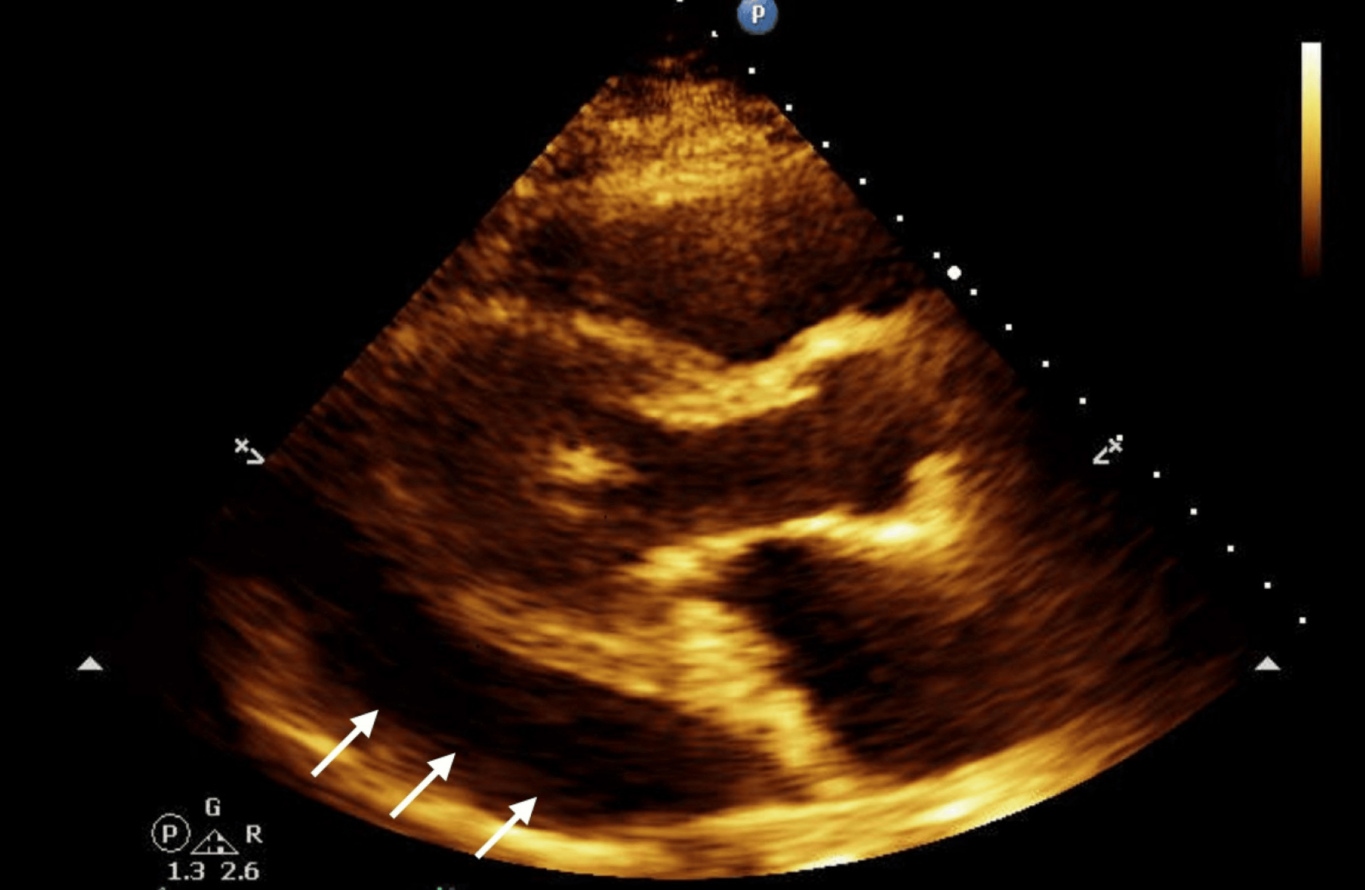
Echocardiogram showing a large posterior pericardial effusion (white arrows) in the parasternal long axis view.

The patient had mild right upper quadrant tenderness on exam. Abdominal ultrasound revealed a shrunken liver with heterogeneous hepatic echotexture and nodular hepatic contour suggesting cirrhosis. One of the initial differential diagnoses included hepatic encephalopathy and he was started on lactulose. Soon, the patient’s hypotension worsened and he was started on minimal vasopressor support. Given this combined with his elevated troponins and a recent cardiac procedure, an echocardiogram was done, and it revealed an ejection fraction of 55-60%; grade I diastolic dysfunction. It also showed a large posterior pericardial effusion adjacent to the left ventricle (Figure 3).

Tamponade was suspected based on worsening hypotension, and the patient was taken for an emergent pericardial window. Intraoperatively, a perforation of the right ventricle was found with a large area of surrounding necrotic and ischemic tissue. There was a significant hemorrhage with the opening of the pericardium, which could not be controlled due to the large area of non-viable cardiac tissue. The patient went into cardiac arrest with pulseless electrical activity. Open cardiac massage was performed but the return of spontaneous circulation could not be achieved and the patient was declared deceased.

## Discussion

The most common cause of atrial fibrillation ablation-related death is cardiac tamponade [2]. The clinical findings in cardiac tamponade include hypotension, increased jugular venous pressure, and muffled heart sounds, also known as Beck's triad. However, these may not be reliable indicators for diagnosis. In the setting of our patient, he only had hypotension; however, he was known to have low blood pressure at baseline and is on midodrine. Sinus tachycardia on ECG with a rate greater than 100 beats per minute is a nonspecific sign of pericardial effusion, particularly cardiac tamponade. In a retrospective study of ECG findings in 241 patients with moderate or large-sized pericardial effusions, sinus tachycardia was present in 43 out of 64 patients, 67% of those with cardiac tamponade, and 41 out of 160, 26% without tamponade. Low voltage of the QRS is another non-specific sign defined as the amplitude of the QRS complexes in the limb leads to be less than 5 mm. Electrical alternans were identified as another ECG sign in 35% of patients with cardiac tamponade and only in 7% without it in the same study of 241 patients [3]. An echocardiogram remains the key diagnostic procedure and should always be done when tamponade is suspected as it is both sensitive and specific. The echocardiographic findings of tamponade include pericardial effusion, diastolic right ventricular collapse, systolic right atrial collapse, a plethoric inferior vena cava with minimal respiratory variation, and an exaggerated respiratory cycle change in mitral and tricuspid valve in-flow velocities [4]. A small pericardial effusion can be treated conservatively, but a significant symptomatic tamponade requires immediate medical attention involving drainage of the effusion with needle pericardiocentesis. In some cases, a catheter drainage or surgical pericardial window may be required to prevent re-accumulation of the effusion.

Recently, a single-center retrospective study was performed to evaluate the role of point-of-care ultrasound (POCUS) in the diagnosis and time to pericardiocentesis of pericardial effusions. A total of 342 patient charts were analyzed and the results were significant for a reduction in time to diagnosis by 39 hours and a reduction in time to pericardiocentesis by 21 hours in those undergoing POCUS as opposed to departmental echocardiography [5]. Hence, POCUS can be a tool used in post-ablation patients to quickly rule in the diagnosis of pericardial effusion and cardiac tamponade.

A large retrospective study with 2467 patients who underwent catheter ablation for atrial fibrillation was conducted to study the incidence and clinical outcomes of cardiac tamponade. Twenty-nine patients were identified to have cardiac tamponade in the study. In regards to timing, 23 among them were noted during the procedure, four during the brief ward stay following the procedure, and only two were found to occur at 10 days and 33 days postoperatively. DCT as in our patient is a rare occurrence, only 0.08% in this study [2].

A systematic review and meta-analysis study done to compare efficacy and safety profiles of high-power, short-duration (HPSD) and low-power, long-duration (LPLD) radiofrequency ablation in atrial fibrillation revealed better outcomes with the HPSD method. The HPSD method reduces esophageal thermal injury (ETI), pulmonary vein reconnection (PVR), and recurrence of atrial fibrillation. The HPSD approach also reduced the procedural time, number of lesions created during pulmonary vein isolation, fluoroscopy time, and post-ablation relapse of atrial fibrillation in one year, improving patient outcomes and safety [6].

The definition of DCT was hypotension or cardiogenic shock requiring pericardial drainage or causing death due to documented pericardial effusion at least one hour following the procedure but attributable to the procedure. In a multi-center study that included a total of 27,921 procedures, DCT was identified in 45 cases. The median time of presentation was found to be 12 days. Patients presented with symptoms to their primary care physicians, emergency physicians, and cardiologists, which further emphasizes the need for awareness. Most patients presented with nonspecific symptoms, including constant thoracic pain, neck or back pain, pain during breathing, dyspnea, dizziness, nausea, fever, peripheral or global edema, nausea, fever, or general malaise. Echocardiography was the mainstay of diagnosis in these patients [7].

Several other cases of DCT following an atrial fibrillation ablation procedure have been reported including a case occurring 61 days postoperatively [8,9]. The underlying mechanism for the development of DCT, as well as the baseline clinical characteristics of patients more likely to develop the complications, remains unclear.

## Conclusions

Pericardial effusion with tamponade requiring intervention is the most fatal complication associated with the ablation procedure. Other vascular complications like bleeding, hematoma, arteriovenous fistula, and femoral pseudoaneurysm are also widely studied. As there are no agreed-upon risk factors contributing to the development of DCT following an ablation, it should be a complication that is discussed with all patients undergoing the procedure. Although rare, given the fatality of the disease process, a surgical backup is essential while performing ablations. Patients should also be encouraged to follow up for four to six weeks postoperatively. DCT should remain a differential diagnosis for physicians when caring for patients presenting even weeks after their procedure with nonspecific symptoms and signs.
